# Stable heterologous expression of biologically active terpenoids in green plant cells

**DOI:** 10.3389/fpls.2015.00129

**Published:** 2015-03-18

**Authors:** N. Kusaira B. K. Ikram, Xin Zhan, Xi-Wu Pan, Brian C. King, Henrik T. Simonsen

**Affiliations:** ^1^Department of Plant and Environmental Sciences, Copenhagen Plant Science Centre, University of CopenhagenCopenhagen, Denmark; ^2^Institute of Biological Sciences, Faculty of Science, University of MalayaKuala Lumpur, Malaysia

**Keywords:** terpenoid production, *Physcomitrella patens*, *Nicotiana tabacum*, plants, *Artemisia annua*

## Abstract

Plants biosynthesize a great diversity of biologically active small molecules of interest for fragrances, flavors, and pharmaceuticals. Among specialized metabolites, terpenoids represent the greatest molecular diversity. Many terpenoids are very complex, and total chemical synthesis often requires many steps and difficult chemical reactions, resulting in a low final yield or incorrect stereochemistry. Several drug candidates with terpene skeletons are difficult to obtain by chemical synthesis due to their large number of chiral centers. Thus, biological production remains the preferred method for industrial production for many of these compounds. However, because these chemicals are often found in low abundance in the native plant, or are produced in plants which are difficult to cultivate, there is great interest in engineering increased production or expression of the biosynthetic pathways in heterologous hosts. Although there are many examples of successful engineering of microbes such as yeast or bacteria to produce these compounds, this often requires extensive changes to the host organism's metabolism. Optimization of plant gene expression, post-translational protein modifications, subcellular localization, and other factors often present challenges. To address the future demand for natural products used as drugs, new platforms are being established that are better suited for heterologous production of plant metabolites. Specifically, direct metabolic engineering of plants can provide effective heterologous expression for production of valuable plant-derived natural products. In this review, our primary focus is on small terpenoids and we discuss the benefits of plant expression platforms and provide several successful examples of stable production of small terpenoids in plants.

## Introduction

Plants are capable of biologically synthesizing a wide array of specialized metabolites. This is reflected in the total number of specialized metabolites reported as well as the structural complexity of individual compounds. These small organic molecules often mediate biological interactions, and may possess bioactive properties that are useful for humans. This makes many specialized plant compounds of high commercial interest to the biotech industry (Cragg et al., 2011). Many of these commercially interesting compounds are terpenoids, and are widely used as flavors, fragrances, pharmaceuticals, nutraceuticals, and industrial chemicals (Berger, 2007; Zwenger and Basu, 2008). Examples of currently marketed drugs originating from a biologically produced terpenoid include artemisinin (sesquiterpenoid), taxol (diterpenoid), and vincristine (meroterpenoid). All of these compounds are highly complex molecules that are commercially obtained from plant starting material. More than 45,000 terpenoids have been identified and this number is increasing (Hamberger and Bak, 2013). They are derived by sequential additions of five carbon isoprene units known as isopentenyl pyrophosphate (IPP). Depending on the number of isoprene units, terpenoids are classified as mono- (2 units), sesqui- (3 units), di-(4 units), tri- (6 units), and so forth. These diphosphate molecules allow for a vast diversity in the core structures when cyclized, forming a backbone upon which secondary modifications often occur, further increasing the diversity. The individual biosynthesis of such unique molecules is often highly complex and involves several classes of enzyme such as terpene synthases, cytochromes P450, and acyl transferases (Drew et al., 2009; Góngora-Castillo et al., 2012; Asada et al., 2013; Drew et al., 2013; Hamberger and Bak, 2013; Weitzel and Simonsen, 2013; Pateraki et al., 2015).

For many bioactive terpenoids of interest to the pharmaceutical, food, or fragrance industries, heterologous expression in engineered organisms is the most commercially sustainable method of production, due to the limited availability from natural sources or the structural complexity of the target compound (Simonsen et al., 2009; Daviet and Schalk, 2010; Nielsen et al., 2014). This however requires extensive knowledge about the native *in planta* biosynthesis of terpenoids in what are often complex metabolic networks, as opposed to linear biosynthetic pathways leading to a single product. Elucidating the biosynthetic route to a target metabolite can take several years of study on just one compound and its biosynthesis (Pickel et al., 2012; Weitzel and Simonsen, 2013; Pateraki et al., 2014, 2015).

In the past 20 years, there have been numerous examples of engineering plants for boosting and altering endogenous isoprenoid metabolism as well as heterologous expression of terpenoid biosynthetic pathways from other species, aiming to improve traits for better disease resistance, weed and pest control, and enhanced ornamental or nutrition properties. See (Table 1) for an overview of the plants and experiments discussed here.

**Table 1:** **Reports of metabolic engineering of plant terpenoids biosynthesis discussed**.

**Overexpressed enzyme(s)**	**Engineered species**	**Gene source(s)**	**Change in terpenoid profile**	**References**
1-deoxy-D-xylulose-5-phosphate synthase (DXS)	*Elaeis guineensis*	*Elaeis guineensis*-fruits	β-carotene ↑	Khemvong and Suvachittanont, 2005
1-deoxy-D-xylulose-5-phosphate synthase (DXS)	*Gingko biloba*	*Gingko biloba*	Ginkgolide ↑	Gong et al., 2006
1-deoxy-D-xylulose-5-phosphate synthase (DXS)	*Lavandula latifolia*	*Arabidopsis thaliana*	Total essential oil ↑	Munoz-Bertomeu et al., 2006
1-deoxy-D-xylulose-5-phosphate synthase (DXS), Deoxyxylulose phosphate reductoisomerase (DXR)	*Salvia sclarea*	*Arabidopsis thaliana*	Aethiopinone ↑	Vaccaro et al., 2014
1-deoxy-D-xylulose-5-phosphate synthase (DXS), Geranylgeranyl diphosphate synthase (GGPPS) 3-hydroxy-3-methylglutaryl-CoA reductase (HMGR)	*Salvia miltiorrhiza*	*Salvia miltiorrhiza*	Tanshinone ↑	Kai et al., 2011
Amorpha-4,11-diene synthase (ADS) Amorpha-4,11-diene oxidase (CYP71AV1) Artemisinic aldehyde Δ 11(13) Double-bond reductase (DBR2)	*Nicotiana tabacum*	*Artemisinia annua*	Amorpha-4,11-diene ↑, artemisinic alcohol ↑, dihydroartemisinic alcohol ↑	Wallaart et al., 2001 Zhang et al., 2011
Amorpha-4,11-diene synthase (ADS) 3-hydroxy-3-methylglutaryl-CoA reductase (HMGR) Farnesyl diphosphate synthase (FPS) Amorpha-4,11-diene oxidase (CYP71AV1)	*Nicotiana benthamiana*	*Artemisinia annua*	Amorpha-4,11-diene ↑ Artemisinic acid-12-β-diglucoside ↑	Van Herpen et al., 2010
Deoxyxylulose phosphate reductoisomerase (DXR)	*Mentha x piperita*	*Mentha* × *piperita*	Total essential oil ↑	Mahmoud and Croteau, 2001
Deoxyxylulose phosphate reductoisomerase (DXR), Amorpha-4,11-diene oxidase (CYP71AV1), Cytochrome P450 reductase (CPR)	*Artemisinia annua*	*Artemisinia annua*	Artemisinin ↑	Xiang et al., 2012
Farnesyl diphosphate synthase (FPS)	*Artemisinia annua*	*Artemisinia annua*	Artemisinin ↑	Han et al., 2006
Geraniol synthase (GES)	*Nicotiana tabacum*	*Valeriana officinalis*	Geraniol ↑	Vasilev et al., 2014
Limonene synthase (LS)	*Nicotiana tabacum*	*Perilla frutescens*	Limonene ↑	Ohara et al., 2003
Limonene-3-hydroxylase (LIM3H), γ-terpinene cyclase (TER) (+)-limonene cyclase1 (LIM) (-)-β-pinene cyclase (PIN)	*Nicotiana tabacum* (Petit Havana, SR1)	*Mentha picata*	(+)-*trans*-isopiperitenol ↑, 1,3,8-*p*-menthatriene ↑, 1,5,8-*p*-menthatriene ↑, *p*-cymene ↑, isopiperitenone ↑	Lücker et al., 2004a
Nerolidol Synthase 1 (FaNES1)	*Arabidopsis thaliana*	*Fragaria ananassa*	Linalool ↑, nerolidol ↑, *E*-8-OH-6,7-dihydrolinalool ↑, *E*-8-OH-linalool ↑	Aharoni et al., 2003
Patchoulol synthase (PTS) Farnesyl diphosphate synthase (FPS)	*Nicotiana tabacum*	*Pogostemon cablin*	Patchoulol ↑	Shuiqin et al., 2006
Patchoulol synthase (PTS) Truncated 3-hydroxy-3-methylglutaryl-CoA-reductase (*t*HMGR)	*Physcomitrella patens*	*Pogostemon cablin Physcomitrella Patens (t*HMGR)	Patchoulol ↑, *Ent*-16-α-OH-kaurene ↓	Zhan et al., 2014
Sclareol synthase 1 and 2 (SsLPPS and SsSS)	*Physcomitrella patens*	*Salvia sclarea*	Sclareol ↑	Pan, 2014
S-linalool synthase (LIS)	*Dianthus caryophyllus* (Carnation)	*Clarkia breweri*	Linalool ↑, *cis*- and *trans*-linalool oxide ↑	Lavy et al., 2002
S-linalool synthase (LIS)	*Lycopersicon esculentum* (Tomato)	*Clarkia breweri*	*S*-linalool ↑, 8-OH-linalool ↑, other volatiles ↑	Lewinsohn et al., 2001
S-linalool synthase (LIS)	*Petunia hybrida* W115	*Clarkia breweri*	S-linalyl-β-glucopyranoside ↑	Lücker et al., 2001
Taxadiene synthase (TXS)	*Arabidopsis thaliana*	*Taxus baccata*	Taxadiene ↑	Besumbes et al., 2004
Taxadiene synthase (TXS)	*Lycopersicon esculentum* Ailsa Craig (Yellow tomato)	*Taxus baccata*	Taxadiene ↑	Kovacs et al., 2007
Taxadiene synthase (TXS)	*Physcomitrella patens*	*Taxus brevifolia*	Taxadiene ↑	Anterola et al., 2009
α/β-santalene (STS)	*Physcomitrella patens*	*Santalum album*	α/β-santalene ↑	Zhan et al., 2014
γ-terpinene cyclase (TER) (+)-limonene cyclase1 (LIM) (-)-β-pinene cyclase (PIN)	*Nicotiana tabacum* (Petit Havana, SR1)	*Citrus x limon*	β-pinene ↑, limonene ↑, γ-terpinene ↑	Lücker et al., 2004b

All the reports have used a strategy based on over expression of the described gene(s) in their experiments. The table was adapted and updated from Lange and Ahkami (2013).

## Overall terpenoid biosynthesis and molecular regulation

Optimization of general isoprenoid metabolism is a well-established strategy to enhance the productivity of terpenoid specialized metabolism. The goal is to increase the amount of carbon going into dedicated isoprenoid precursors, pushing metabolic flux through to the final biologically active specialized metabolite. The engineering strategy often involves over-expression of the 2-C-methyl-D-erythritol-4-phosphate (MEP) and mevalonic acid (MVA) pathway rate-limiting genes. For the MEP pathway, these enzymes include 1-deoxy-D-xylulose-5-phosphate synthase (DXS), 1-Deoxy-D-xylulose 5-phosphate reductoisomerase (DXR), and (*E*)-4-hydroxy-3-methylbut-2-enyl diphosphate reductase (HDR). For the MVA pathway, the rate limiting step is generally considered to be 3-hydroxy-3-methyl-glutaryl-CoA reductase (HMGR) (Enfissi et al., 2005; Carretero-Paulet et al., 2006; Munoz-Bertomeu et al., 2006; Ohto et al., 2009; Song et al., 2012; Paddon et al., 2013). Overexpression of this step has been shown both in microorganisms and in plants to increase terpenoid production (Wang et al., 2012; Paddon et al., 2013). Other approaches target enrichment of the direct isoprenoid precursor pool through up-regulation of the prenyltransferases geranyl diphosphate synthase (GPS), farnesyl diphosphate synthase (FPS), or geranylgeranyl diphosphate synthase (GGPPS) (Takahashi et al., 2007; Bruckner and Tissier, 2013). Additional strategies include knockdown of biosynthetic pathways that compete for precursor molecules, such as knockdown of squalene synthase or gibberellic acid synthesis (Shuiqin et al., 2006; Engels et al., 2008; Wu and Chappell, 2008). However, knockdown can also have unintended negative effects on pathway regulation resulting in altered plant growth (e.g., in rice) and final yield of the desired product (Manavalan et al., 2012; Zhan et al., 2014). Finally engineering transcription factors or promoters controlling key biosynthetic genes is an emerging field, increasing knowledge of promoter design, DNA motifs important for transcription factor binding to promoters, and which transcription factors are active. For artemisinin biosynthesis in the plant *Artemisia annua* it was found that high and low producing varieties mainly differ in the promoter region of the key gene DBR2 (Yang et al., 2015). This step controls production of either artemisinin or arteannuien B in the plants, and the two promoters have different binding sites for different transcription factors illustrating the importance of this regulation. The choice of promoters for biotechnological production is well-described and numerous promoters are characterized for not only microbial biotechnology, but also for plant biotechnology (Venter, 2007), and modifications of these have become a key approach in metabolite engineering, including the use of synthetic promoters (Blount et al., 2012).

**Figure 1:**
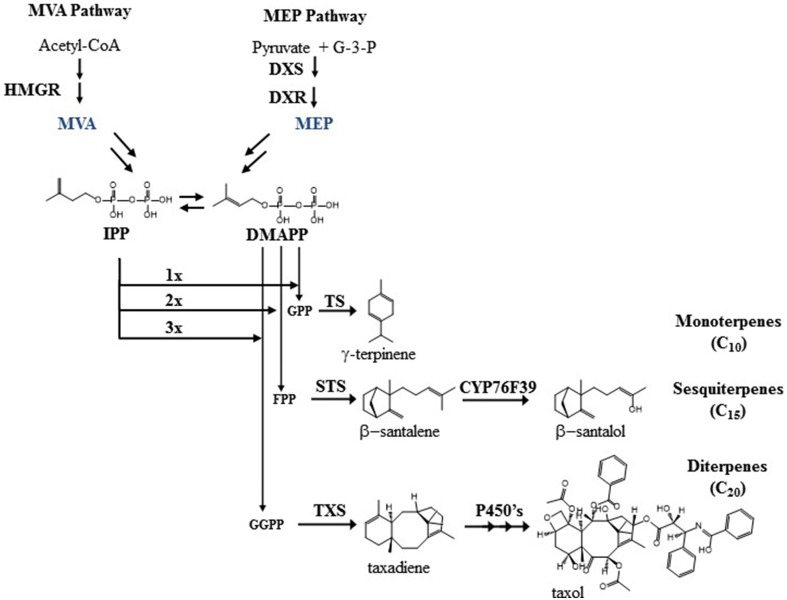
**The figure show the overall MVA and MEP pathway, along with examples of mono-, sesqui-, and diterpenoids biosynthesis also described in the text**.

Looking at general isoprenoid metabolism in more detail, terpenoid biosynthesis starts from the universal building block IPP (Figure 1). Two compartmentalized pathways, the cytosolic MVA pathway and the plastidic MEP pathway, are responsible for IPP biogenesis (Rohmer et al., 1993). In contrast to *Saccharomyces cerevisiae* and *Escherichia coli*, plants (including the primitive non-vascular land plant *Physcomitrella patens*) have both pathways for terpenoid biosynthesis. In the MVA pathway, the NADPH-dependent HMGR is considered as the rate-limiting enzyme that has a global control upon the metabolic flux (Stermer et al., 1994; Chappell et al., 1995; Nieto et al., 2009). In the MEP pathway, the first step is the biosynthesis of 1-deoxy-D-xylulose-5-phosphate (DXP) by DXS. This is a rate limiting step together with the second step, where DXP is reduced by DXR using NADPH as a cofactor to 2-C-methyl-D-erythritol 4-phosphate (Figure 1). In the MEP pathway, HDR has been shown to play a major role in controlling plastid terpenoid biosynthesis along with DXS and DXR (Rodriguez-Concepcion et al., 2004).

In the beginning of both pathways, the isomerization of IPP to DMAPP is catalyzed by isopentenyl pyrophosphate isomerase (IDI), which plays a crucial role in plants. When IDI was disrupted in either *Nicotiana benthamiana* (Page et al., 2004) or *Arabidopsis thaliana* (Okada et al., 2008) up to 80% reduction in chlorophylls, phytosterols, and carotenoids was observed.

As the universal precursors of terpenoid biosynthesis, IPP and DMAPP are polymerized by prenyltransferases such as GPS, FPS, and GGPPS, followed by terpene synthases (TPS) that utilize GPP, FPP, or GGPP as substrates. GPS and GGPPS are mainly found in the plastids and FPS is found in the cytosol. As the precursor pathways and metabolite pools are generally compartmentalized in plants, so follows the specialized metabolism with monoterpene synthases and diterpene synthases working in the plastid whilst sesquiterpene synthases are usually found in the cytosol. However, some cases of ambiguous sub-cellular location of these enzymes contribute to a more complex pattern of terpenoid biosynthesis. For example, in *Lithospermum erythrorhizon*, GPS is found in the cytosol instead of the chloroplast, and in rice and wild tomato, FPS is found in both the chloroplast and the cytosol. The chloroplastic enzyme in wild tomato produces *Z,Z*-FPP instead of *E,E*-FPP that is the product of the cytosolic enzyme (Sommer et al., 1995; Sanmiya et al., 1999; Sallaud et al., 2009). Likewise, some terpene synthases are found in unexpected subcellular locations, which can further enhance chemical diversity. Examples are the dual plastid/mitochondrial localized FaNES2 from strawberry, the monoterpene synthase α-terpineol synthase from *Magnolia*, and the monoterpene synthase FvPIN from strawberry that are localized to the cytosol (Aharoni et al., 2004; Lee and Chappell, 2008). The sesquiterpene synthase for santalene and bergamotene from wild tomato is localized to the plastids (Sallaud et al., 2009). In contrast, the essential oil producing plant sandalwood has santalene and santalol biosynthesis localized in the cytosol of cells in the heartwood where a santalene synthase (STS) cyclizes FPP followed by a hydroxylation by CYP76F39 yielding santalol and bergamotene (Diaz-Chavez et al., 2013). The product profile is slightly different between the STS from tomato and sandalwood, and so is the stereochemistry of the compounds. Collectively, the different locations give a more dynamic picture of terpenoid biosynthesis than the classical descriptions, and offer additional biochemical routes to enhance the chemical diversity in future bioengineering projects.

## Production of heterologous terpenoids in plants

There are many examples of successful heterologous expression of terpene biosynthetic genes and increased production levels of terpenoids, particularly via microbial hosts. However, most complex plant-derived terpenoids such as taxol and artemisinin require plant cytochrome P450 enzymes, which are often difficult to express in microbial cells due partially to membrane associations and subcellular targeting, as well as post-translational modifications and insolubility. Thus, complex engineering strategies for expression in microbial hosts are required in order to obtain the complete biosynthesis of the target molecule. Attempts at using plant cells, as heterologous hosts involving cytochromes P450 are less widespread, mainly because plant transformation techniques are more complex, are not available for many species, require much more time to recover transgenic organisms, and plant growth rates are typically slower than in microbial systems. However, in part due to their native cytochrome P450-related biosynthetic pathways, plant cells may be more desirable to engineer plant pathways compared to the microbial options. Several expression studies using plant cells for heterologous terpenoid production have been carried out, and we will focus mainly on examples in *Nicotiana* spp., *A. thaliana*, and *P. patens* (see Table 1).

Expression of amorphadiene synthase from *Artemisia annua* in *N. tabacum* resulted in the production of amorphadiene, yielding 0.2–1.7 ng/g (Wallaart et al., 2001). Adding two additional artemisinin biosynthetic genes yielded 4 mg/g fresh weight of amorphadiene in leaves and 0.01 mg/g dry weight of artemisinic alcohol (Zhang et al., 2011). Following the successful amorphadiene production in *N. tabacum*, *N. benthamiana* was then considered as another production platform for artemisinin precursors. The study aimed to produce artemisinic acid by combined transient expression of amorphadiene synthase targeted to the mitochondria, followed by mitochondrial FPS and cytosolic HMGR co-expressed using a ribosomal-skip construct. These genes were co-infiltrated with CYP71AV1 (amorphadiene oxidase) and the plants produced artemisinic acid-12-β-diglucoside at 39.5 mg/kg fresh weight instead of artemisinic acid. It has been suggested that the expressed artemisinic acid was further modified by a native glycosyl transferase into artemisinic acid-12-β-diglucoside (Van Herpen et al., 2010; Ting et al., 2013).

Another example explored stable heterologous expression in *Nicotiana* of the monoterpenoid geraniol synthase from *Valeriana officinalis* (*VoGES*) and compared several *N. tabacum* and *N. benthamiana* systems. This study compared productivity of stable and transient expression and different growth conditions including whole plants and cell cultures. The authors observed highest geraniol yield in stable transgenic plants grown in the greenhouse, and 10-fold reduction when plants were grown *in vitro*. However, they also conclude that cell suspension cultures were superior to either hairy root cultures or greenhouse plants due to the relatively high biomass yield and short cultivation time (9 days compared to 6–9 weeks) and suggest that cell cultures could be the most economically viable option for large scale production (Vasilev et al., 2014).

Production of patchoulol has also been shown in tobacco at 0.030 mg/g and volatile emission of 50–100 ng/h fresh weight in the leaves. It was shown that redirecting the production of sesquiterpenoids from the cytosol to the plastids increased the overall yield by 40,000 fold (Shuiqin et al., 2006).

Another well-researched plant host for gene overexpression is *Arabidopsis thaliana*. Taxadiene synthase (TXS) from *Taxus baccata* was expressed in *Arabidopsis*, which led to production of taxadiene, a key step of paclitaxel/Taxol biosynthesis. Taxadiene accumulation was reported to be about 20 ng/g dry weight produced in both seedlings and leaves from the transgenic lines (Besumbes et al., 2004). Besides *Arabidopsis*, TXS has also been expressed in yellow tomato, where redirecting GGPP from making carotenoids into producing taxadiene resulted in 160 mg from 1 kg of freeze dried fruit (Kovacs et al., 2007).

The moss *Physcomitrella patens* is an attractive alternative for production of plant biopharmaceuticals due to the fact that it can be scaled up in liquid cultures in large bioreactors and genetically modified using homologous recombination. Mosses have a simple terpenoid profile, which facilitates a clean metabolic background. Despite this simple profile, *P. patens* produces large quantities of endogenous diterpenes (Von Schwartzenberg et al., 2004). A combination of these factors makes moss an attractive host for heterologous terpenoid biosynthesis. For example, the expression of TXS in *Physcomitrella patens* produces taxadiene without any phenotypic change, making it a promising host for the production of paclitaxel (Anterola et al., 2009). In addition, recently published work demonstrated successful production of two important sesquiterpenoids for the fragrance industry in moss, patchoulol and β-santalene, with productivity up to 1.3 mg/g and 0.039 mg/g dry weight, respectively (Zhan et al., 2014). In this study, overexpression of a truncated HMGR lacking a regulatory domain either from moss itself or from yeast gave an increase in patchoulol levels. Another important diterpenoid for the fragrance industry, sclareol, was also recently produced in moss, with yield that yielded 2.84 mg/g dry weight in the highest producing moss line (Pan, 2014).

### Transgenic events negatively affected primary metabolism

Metabolic engineering of terpenoid biosynthesis pathways in plants could have physiological and ecological costs, as demonstrated by growth retardation following DXR over-expression in peppermint for the high yield of essential oil (Mahmoud and Croteau, 2001). Similar issues have been observed in *Arabidopsis* and tomato, where heterologous expression of taxadiene synthase (TXS), linalool/nerolidol synthase (FaNES1), phytoene synthase (PSY), or geraniol synthase (GES) negatively affected the biosynthesis of primary metabolites (Busch et al., 2002; Aharoni et al., 2003; Besumbes et al., 2004; Davidovich-Rikanati et al., 2007). One explanation for these phenotypes might be due to a toxic effect in plant cells. A more detailed study in maize, which explored heterologous biosynthesis of (*E*)-β-caryophyllene and α-humulene, suggested that the costs such as compromised seed germination, plant growth, and yield outweighed its potential benefits such as repelling root herbivores (Robert et al., 2013). Silencing of (*E*)-4-hydroxy-3-methylbut-2-enyl diphosphate synthase (HDS) and isopentenyl/dimethylallyl diphosphate synthase (IDS) also negatively affect the level of chlorophyll and carotenoids in leaves compared to controls (Page et al., 2004). Two reasonable possibilities could explain such costs: depletion of precursor for the primary metabolites, and the toxicity of desired products (Fray et al., 1995; Aharoni et al., 2003).

## Production hosts—microbial vs. plant platforms

The most popular hosts used for the production of natural products, including terpenoids, are *E. coli* and *S. cerevisiae*. These two microorganisms are well-established and there is a large body of knowledge and genetic resources for them, including whole-cell computer models (Atlas et al., 2008). Versatile molecular cloning tools and accessibility of scale-up in industry are additional advantages of these microbial platforms. *E. coli* has been used for production of artemisinin precursors. A modified *E. coli* strain together with a superior fermentation process was able to accumulate more than 25 g/L amorpha-4,11-diene (precursor of artemisinic acid and artemisinin) in the culture (Tsuruta et al., 2009). However, the challenges of expressing plant cytochromes P450 (e.g., CYP71AV1 for artemisinic acid synthesis) makes *E. coli* a less suitable host for complex terpenoid production, due to the need of these enzymes for further product modification and precise membrane associations. In such cases, the baker's yeast *S. cerevisiae* has been used as a eukaryotic expression host, especially when only one cytochrome P450 is involved. By introducing the genes responsible for artemisinic acid synthesis, including CYP71AV1 and its redox partner POR (cytochrome P450 oxido-reductase), engineered yeast is capable of producing high titers (more than 100 mg/l) of artemisinic acid (an intermediate in the artemisinin biosynthesis pathway). This level of expression is significantly higher than in *Artemisia annua*, the natural producer of artemisinin (Ro et al., 2006). The commercialization of yeast-based pharmaceuticals such as artemisinin (Paddon et al., 2013), the forskolin precursor manoyl oxide (Nielsen et al., 2014; Pateraki et al., 2014), and some fragrance compounds is imminent, or already available.

Metabolic engineering of plant metabolism is far more complicated and time consuming as compared to the established microbial systems. In depth understanding of the metabolic system and regulatory complexity would be very helpful to manipulate the system without disrupting other metabolic processes within the plant. Compared to microbial platforms, plants are generally not regarded as a first-choice platform for terpenoid production due to the more complicated metabolic network, difficulty of stable transformation, longer growth cycles, and challenges in industrial scale-up of cell cultures. However, a major limitation of many microbial expression systems, in particular the use of bacterial systems, is that they are unable to carry out the necessary post-translation modification essential for correct folding and subsequent protein function. As such, many of these microbial systems produce eukaryotic enzymes that are not functionally active. Additionally, as photoautotrophic organisms, plants have their own advantages including the capability of utilizing carbon dioxide as a carbon source, light-driven biosynthesis, and growth in open fields or greenhouses. This means that plants could potentially be grown in more environmentally friendly ways and have a smaller carbon footprint than industrial bioreactors for yeast and bacteria. Monocots (duckweed, maize), dicots (alfalfa, *N. benthamiana*), and bryophytes (*P. patens*) are used to produce therapeutic proteins (Faye and Gomord, 2009; Buttner-Mainik et al., 2011; Bach et al., 2014a,b). As described previously, *P. patens* can also be used to produce both sesqui- and diterpenoids and is readily scaled-up in illuminated bioreactors (Anterola et al., 2009; Pan, 2014; Zhan et al., 2014). Terpenoid production in algae is also an attractive option but much work needs to be done before an industrial application is available (Lohr et al., 2012). Another advantage of heterologous expression in plant platforms is that increasing endogenous terpenoid products in the native plants may improve the nutritional value (such as carotenoids, pro-vitamin A) of food crops as well as enhancing plant fitness by increasing the resistance to herbivores, pathogens, or pests (Ye et al., 2000; Beale et al., 2006; Munoz-Bertomeu et al., 2006; Beyer, 2010).

## Metabolic regulation for successful plant terpenoid engineering

Due to the importance of terpenoids in growth, development, and survival, plants have evolved a complicated network to regulate terpene biosynthesis in different layers (Chappell, 1995; McGarvey and Croteau, 1995). In general, for the MEP pathway up or down-regulation of the rate-limiting DXS and DXR genes will positively or negatively affect the overall biosynthesis of plastidial terpenoids including carotenoids, chlorophylls as well as heterologous terpenes (Estevez et al., 2001; Carretero-Paulet et al., 2006). These two genes are regulated by light, developmental cues and biotic elicitors at transcriptional and post-transcriptional levels (Walter et al., 2000; Okada et al., 2007; Cordoba et al., 2009; Han et al., 2013). For instance, the DXS transcript was at a higher level in the young leaves and inflorescences of tomato as well as the ripening fruit, but not in the root indicating a light inducible regulation (Lois et al., 2000). HMGR, the key regulatory step in the MVA pathway is regulated in response to a variety of developmental and environmental stress factors (Choi et al., 1994; Weissenborn et al., 1995). HMGR is regulated at the transcriptional, translational, and post-translational levels (Stermer et al., 1994; Nieto et al., 2009).

Efforts have been made to enhance the production of terpenoid bioactive compounds by modulating the MEP and MVA activity to boost production. This is shown in the production of essential oil in mint that gives up to 40% increase of essential oils (Lange and Croteau, 1999) due to an increase in deoxyxylulose phosphate reductoisomerase (DXR) activity by overexpression of the DXR gene. Overexpression of DXS from *Arabidopsis* in spike lavender also led to an increased yield of essential oils with 3.5 fold in leaves and 6 fold in flowers compared to controls (Munoz-Bertomeu et al., 2006). A similar situation can be observed on the production of ginkgolide in *Ginkgo biloba* (Gong et al., 2006), β-carotene in *Elaeis guineensis* (Khemvong and Suvachittanont, 2005), and carotenoid in *A. thaliana* (Estevez et al., 2001).

Combining strategies of boosting general isoprenoid metabolism as well as overexpression of genes for specialized metabolism has also given good results. Transgenic *Artemisia annua* expressing three functional artemisinin-related genes, DXR, CYP71AV1, and POR showed higher production levels of artemisinin than the wild type (Xiang et al., 2012). Production was also increased by overexpression of FPS with up to threefold increase of artemisinin (Han et al., 2006). Co-expression of FPS and amorpha-4,11-diene synthase (ADS) in the plastids in tobacco likewise increases accumulation of the artemisinin precursor amorpha-4,11-diene (Shuiqin et al., 2006).

In *Salvia sclarea* hairy root cultures, Vaccaro et al. demonstrated a successful independent overexpression of DXS and DXR from *Arabidopsis*. The expressed genes enhanced the biosynthesis of diterpenes resulting in a 2.5 fold increase of aethiopinone for DXS and 4.6 fold for DXR (Vaccaro et al., 2014). Previous work by Kai et al. showed that overexpressing DXS, GGPPS, and HMGR in *Salvia miltiorrhiza* hairy root led to accumulation of tanshinone at about 4.7 fold higher than the control (Kai et al., 2011).

## Perspectives

Engineering of terpenoid biosynthesis in plants introduces several additional challenges compared to microbial hosts. However, plant metabolic engineering also provides additional benefits and opportunities. Knowledge of plant metabolic networks and metabolic trafficking, at both the biochemical and cellular level, has increased. This, along with the fast development of next generation sequencing and novel genetic modification techniques, provide tremendous tools for plant engineering in scales that just a few years ago would have been a daunting task. For example, advances in plant molecular and synthetic biology have greatly expanded the capacity for targeted genome editing in higher plants. The tools provided by zinc finger nucleases, TALENs, and the CRISPR/Cas9 reagents greatly facilitate generation of targeted deletions and insertions of foreign DNA. The moss *Physcomitrella* has been a popular model plant for targeted genome editing for nearly 20 years due to its native ability for efficient homologous recombination, and even with the advances being made with genome editing in higher plants, moss remains well-positioned to be a model for studying terpenoid biosynthesis as well. The molecular tools for algae and cyanobacteria are also growing, and these organisms may prove to be good hosts for terpenoid engineering. Thus, the benefits of using plant cells as chemical factories for complex plant derived compounds remains an attractive alternative to microbial systems as the technological gap between the platforms becomes smaller. Overall, in many cases, the benefits of plants as a production system can outweigh the slower generation time and other challenges, and resources for engineering terpenoid metabolism directly *in planta* should continue to be developed. The increased awareness and acceptance of plants as small molecule production hosts combined with the generation of tools and knowledge that can be used in future plant engineering projects will lead to further success, bringing us closer to more examples of industrial scale green cell factories for terpenoids and other valuable specialized metabolites.

### Conflict of interest statement

The authors declare that the research was conducted in the absence of any commercial or financial relationships that could be construed as a potential conflict of interest.
